# Clinical and genetic characteristics of patients with Doose syndrome

**DOI:** 10.1002/epi4.12417

**Published:** 2020-07-23

**Authors:** Nodoka Hinokuma, Mitsuko Nakashima, Hideyuki Asai, Kazuyuki Nakamura, Shinjiro Akaboshi, Masataka Fukuoka, Masami Togawa, Shingo Oana, Koyo Ohno, Mariko Kasai, Chikako Ogawa, Kazuna Yamamoto, Kiyohito Okumiya, Pin Fee Chong, Ryutaro Kira, Shumpei Uchino, Tetsuhiro Fukuyama, Tomoe Shinagawa, Yohane Miyata, Yuichi Abe, Akira Hojo, Kozue Kobayashi, Yoshihiro Maegaki, Nobutsune Ishikawa, Hiroko Ikeda, Masano Amamoto, Takeshi Mizuguchi, Kazuhiro Iwama, Toshiyuki Itai, Satoko Miyatake, Hirotomo Saitsu, Naomichi Matsumoto, Mitsuhiro Kato

**Affiliations:** ^1^ Department of Pediatrics Showa University School of Medicine Tokyo Japan; ^2^ Department of Biochemistry Hamamatsu University School of Medicine Hamamatsu Japan; ^3^ Department of Human Genetics Yokohama City University Graduate School of Medicine Yokohama Japan; ^4^ Department of Pediatrics Yamagata University Faculty of Medicine Yamagata Japan; ^5^ Department of Pediatrics Tottori Medical Center Tottori Japan; ^6^ Shizuoka Institute of Epilepsy and Neurological Disorders Shizuoka Japan; ^7^ Department of Pediatrics Tottori Prefectural Central Hospital Tottori Japan; ^8^ Department of Pediatrics Tokyo Medical University Tokyo Japan; ^9^ Division of Child Neurology Institute of Neurological Sciences Faculty of Medicine Tottori University Yonago Japan; ^10^ Department of Developmental Medical Sciences Graduate School of Medicine The University of Tokyo Tokyo Japan; ^11^ Department of Pediatrics Nagoya University Graduate School of Medicine Aichi Japan; ^12^ Department of Pediatrics Teikyo University School of Medicine Tokyo Japan; ^13^ Department of Pediatrics and Child Health Kurume University School of Medicine Fukuoka Japan; ^14^ Department of Pediatric Neurology Fukuoka Children's Hospital Fukuoka Japan; ^15^ Department of Neuropediatrics Tokyo Metropolitan Neurological Hospital Tokyo Japan; ^16^ Department of Pediatrics The University of Tokyo Tokyo Japan; ^17^ Department of Pediatrics Shinshu University School of Medicine Matsumoto Japan; ^18^ Department of Pediatrics Aomori City Hospital Aomori Japan; ^19^ Department of Pediatrics Kyorin University Faculty of Medicine Tokyo Japan; ^20^ Department of Pediatrics Saitama Medical University Moroyama Japan; ^21^ Division of Neurology National Center for Child Health and Development Tokyo Japan; ^22^ Department of Pediatrics Hiroshima University Hospital Hiroshima Japan; ^23^ Kitakyushu City Yahata Hospital Pediatric Emergency/Children’s Medical Center Fukuoka Japan

**Keywords:** comorbidity, Doose syndrome, *HNRNPU*, *SLC6A1*, *STS*

## Abstract

**Objective:**

To elucidate the genetic background and genotype‐phenotype correlations for epilepsy with myoclonic‐atonic seizures, also known as myoclonic‐astatic epilepsy (MAE) or Doose syndrome.

**Methods:**

We collected clinical information and blood samples from 29 patients with MAE. We performed whole‐exome sequencing for all except one MAE case in whom custom capture sequencing identified a variant.

**Results:**

We newly identified four variants: *SLC6A1* and *HNRNPU* missense variants and microdeletions at 2q24.2 involving *SCN1A* and Xp22.31 involving *STS*. Febrile seizures preceded epileptic or afebrile seizures in four patients, of which two patients had gene variants. Myoclonic‐atonic seizures occurred at onset in four patients, of which two had variants, and during the course of disease in three patients. Variants were more commonly identified in patients with a developmental delay or intellectual disability (DD/ID), but genetic status was not associated with the severity of DD/ID. Attention‐deficit/hyperactivity disorder and autistic spectrum disorder were less frequently observed in patients with variants than in those with unknown etiology.

**Significance:**

MAE patients had genetic heterogeneity, and *HNRNPU* and *STS* emerged as possible candidate causative genes. Febrile seizures prior to epileptic seizures and myoclonic‐atonic seizure at onset indicate a genetic predisposition to MAE. Comorbid conditions were not related to genetic predisposition to MAE.

**Key Points**

- The genetic background of epilepsy with myoclonic‐atonic seizures or myoclonic‐astatic epilepsy (MAE) is heterogeneous and mainly unknown
- Four of 29 patients with MAE showed a pathogenic variant or a microdeletion
- Two genes, *HNRNPU* and *STS*, were newly identified as possibly responsible for MAE
- Febrile seizures prior to epileptic seizures or myoclonic‐atonic seizures at onset might indicate a certain genetic etiology for MAE

## 1 INTRODUCTION

Epilepsy with myoclonic‐atonic seizures, also known as myoclonic‐astatic epilepsy (MAE) or Doose syndrome, was first reported as “centrencephalic myoclonic‐astatic petit mal” by Doose et al^1^ in 1970. MAE, which accounts for 1%‐2.2% of childhood‐onset epilepsy cases, is characterized by normal development before seizure onset, which generally occurs between 7 months and 6 years of age. Patients commonly exhibit myoclonic, myoclonic‐atonic, and generalized tonic‐clonic seizures (GTCs), as well as tonic seizures with generalized onset, atypical absences, and nonconvulsive status epilepticus.^2^ Interictal EEG is often normal at disease onset, but patients almost always develop parietal‐dominant rhythmic theta activity and sometimes a posterior 4 Hz rhythm that is attenuated by eye opening.^3^ Although generalized seizure activity is characteristic in MAE, pseudofoci of electrical activity may also be seen in EEGs.^3^ Although sodium valproate (VPA), ethosuximide (ESM), benzodiazepines (BZPs), lamotrigine (LTG), and a ketogenic diet (KD) have been found to be effective in the treatment of MAE, seizure prognosis varies by case. Between 50% and 80% of patients respond to treatment, and their seizures disappear within 3 years from the start of treatment, while other patients exhibit intractable seizures and depressed intellectual development.^4^, ^5^

Given that the male‐to‐female ratio of MAE is 2‐3:1 and that 14%‐32% of patients have a family history of epileptic seizures or EEG abnormalities, researchers have long suspected genetic predisposition.^1^, ^5^ So far, 11 genes, *SCN1A*,^6^ *SCN1B*,^7^ *CACNA1H*,^8^ *SLC2A1*,^9^ *GABRG2*,^10^ *CHD2*,^10^ *SLC6A1*,^11^ *STX1B*,^12^ *GABRB3*,^13^ *SYNGAP1*,^14^ and *WDR45*,^15^ have been reported to be causative for MAE. This suggests that MAE is highly heterogeneous, as Doose originally predicted. The causative genes show autosomal inheritance with the exception of *WDR45* on Xp11.23, for which variants show female predominance. Thus, the male predominance of MAE remains unexplained. In this study, we aimed to identify the genetic causes of MAE and to establish the differences in clinical features between patients with a genetic predisposition and those with an unknown etiology.

## 2 METHODS

### 2.1 Patients and variant analysis

Thirty‐one patients were assessed for eligibility, and 29 patients with MAE participated in this study (Figure S1), which was conducted as part of a nationwide Japanese gene analysis. We retrospectively collected clinical information from all patients including clinical history, seizure semiology, EEG and MRI findings, laboratory test results, and antiepileptic drug (AED) treatment records including drug effectiveness, psychomotor development records, and comorbidities. The diagnosis of MAE was conducted based on the following clinical features and EEG findings^16^, ^17^: (a) unremarkable antecedent and birth history; (b) onset of seizures between 6 months and 6 years of age; (c) myoclonic, atonic, myoclonic‐atonic, absence, or tonic‐clonic seizures; and (4) EEG that was often initially normal followed by a biparietal theta rhythm and generalized spike‐and‐wave or polyspike‐and‐wave activity. Although a recent definition of MAE by the International League Against Epilepsy (ILAE) lists the presence of myoclonic‐atonic seizures as a requirement (https://www.epilepsydiagnosis.org), most studies on MAE have applied the original definition by Doose.^1^, ^2^ In this study, we used the original seizure semiology proposed by Doose to compare our cohort with those of previous studies. We included patients with tonic seizures or focal/multifocal EEG spikes because tonic seizures have been reported to develop late in the course of cases with an unfavorable outcome,^16^, ^18^ and multifocal EEG spikes have been observed in MAE patients.^17^, ^18^ We excluded patients with the following diagnoses: benign myoclonic epilepsy in infancy, Dravet syndrome, Lennox‐Gastaut syndrome, atypical childhood epilepsy with centrotemporal spikes, glucose transporter disorders, and progressive myoclonus epilepsies (https://www.epilepsydiagnosis.org). Patients with structural brain defects that might explain their epilepsy, such as lissencephaly, focal cortical dysplasia, and white matter abnormalities, were excluded via brain MRI conducted at 1.5 T or 3 T. AED effectiveness was classified into four categories in terms of the effect on seizures and EEG, respectively: “disappeared/normalized,” “decreased (the frequency of seizures was less than 50%)/improved (typical findings, such as a suppression‐burst pattern or hypsarrhythmia, disappeared but epileptic discharges continued),” “no changes/no changes,” and “worsened/unknown”. The attending physicians or clinical psychologists in each hospital administered developmental or intellectual assessment tests, mainly the Wechsler Intelligence Scale for Children‐Fourth edition (WISC‐IV), Japanese Enjoji Developmental scale, and the Kyoto Scale of Psychological Development, according to the age and developmental level of the patient at the last visit or at least one year after the onset of seizures.

### 2.2 Gene analysis

We analyzed genetic material from the 29 patients and each set of parents. Genomic DNA was extracted using QuickGene‐610L (Fujifilm) according to the manufacturer's instructions. DNA from five patients was enriched using the Nextera rapid capture custom enrichment kit (Illumina) for original target regions of known and candidate genes related to developmental and epileptic encephalopathy (DEE;154 genes in Patients 23 and 29, 198 genes in Patients 2, 6, 7, and 14) and was sequenced using the MiSeq system (Illumina) with the MiSeq Reagent Kit v2, with 500 cycles and paired‐end sequencing. Detected variants were analyzed using VariantStudio (Illumina). For whole‐exome sequencing (WES), DNA from 28 patients was captured using the SureSelect Human All Exon V5 Kit (Agilent Technologies) and sequenced using HiSeq2500 (Illumina) with 101‐bp paired‐end reads. In Patient 2, a causative variant was identified using the custom capture method. Exome data processing, variant calling, and annotation were performed as previously described.^19^ Pathogenicity prediction was performed using SIFT (http://sift.bii.a‐star.edu.sg/), Polyphen‐2 (http://genetics.bwh.harvard.edu/pph2/), LRT (http://www.genetics.wustl.edu/jflab/lrt_query.html), MutationTaster (http://www.mutationtaster.org/), S‐VAR (http://p4d‐info.nig.ac.jp/s‐var/), HGMD (http://www.hgmd.cf.ac.uk/ac/index.php), FATHMM (http://fathmm.biocompute.org.uk/cancer.html), GERP++ (http://mendel.stanford.edu/sidowlab/downloads/gerp/index.html), PhyloP (http://compgen.bscb.cornell.edu/phast/), and CADD (https://cadd.gs.washington.edu). Copy number variations (CNVs) were examined using WES data with two algorithms: the eXome‐Hidden Markov Model (XHMM)^20^ and a program developed by Nord et al^21^ that uses the relative depth of coverage ratios. Single nucleotide or copy number variants and their familial segregation were confirmed using Sanger sequencing (ABI 3130xl Genetic Analyzer, Applied Biosystems) or quantitative polymerase chain reaction analysis (PCR; Rotor Gene 6000 Real Time Analyzer, Corbett Life Science), respectively. All gene variants were classified according to the ACMG variant classification criteria.^22^

### 2.3 Standard protocols, registrations, and patient consent

Clinical information and peripheral blood samples were acquired from all patients and their parents after obtaining written informed consent. The study protocol was approved by the Institutional Review Boards of Yamagata University Faculty of Medicine, Showa University School of Medicine, and Yokohama City University School of Medicine.

### 2.4 Data analysis

We analyzed the collected clinical information using descriptive statistics. We counted the number of patients who had received each AED and calculated the ratio of patients in whom the AED was effective to the total number of patients. We classified the patients into two groups: a seizure‐free group, in which the patients were seizure‐free for at least 6 months and had not experienced seizure recurrence at the last visit, and the intractable seizure group, comprising patients in whom more than two AEDs were ineffective and seizures had persisted at the last visit. We compared the patients according to category and considered the relationships between the clinical and genetic features.

## 3 RESULTS

### 3.1 Identification of causative genes

**FIGURE 1:**
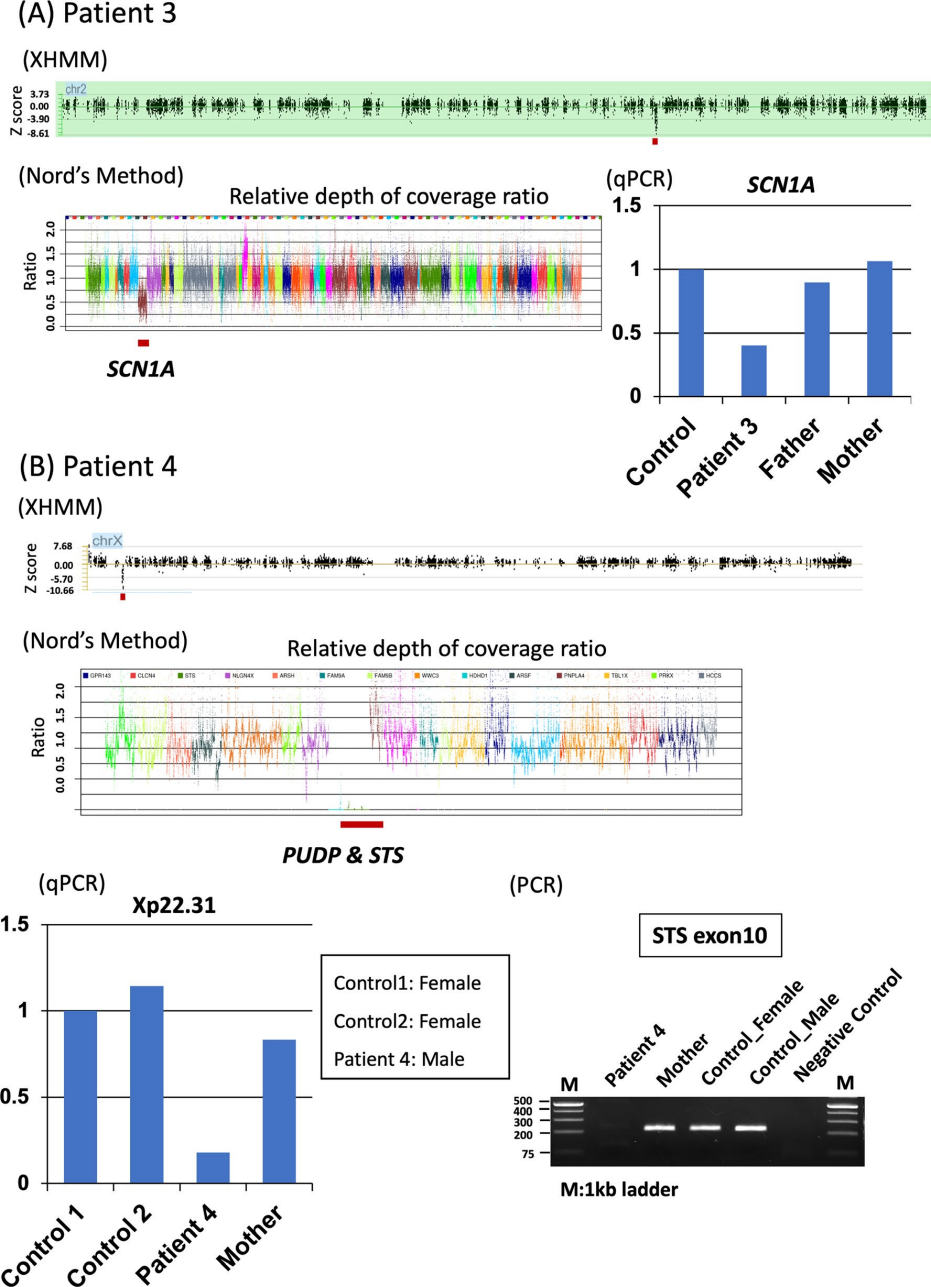
Small pathogenic CNVs detected by WES‐based programs. Results of CNV analyses by XHMM and Nord's method and quantitative PCR (qPCR) in Patients 3 (A) and 4 (B). Results of PCR for exon 10 of the *STS* gene in Patient 4 (B). Thick red bars represent calls for copy number losses in each method. Patient 3 showed a de novo 588.7‐kb deletion involving *SCN1A* at 2q24.3, which was confirmed by qPCR for SCN1A. Patient 4 showed a de novo hemizygous 299.9‐kb deletion involving *HDHD1* and *STS* at Xp22.31, which was confirmed by qPCR and PCR methods using primer pairs for exon 10 of *STS*

We newly identified de novo variants in two DEE‐related genes (*SLC6A1* and *HNRNPU*) and two microdeletions on 2q24.3 and on Xp22.31. Four patients showed pathogenic variants in this MAE cohort (Table S2). The *SLC6A1* and *HNRNPU* variants in Patients 1 and 2, respectively, are novel and neither were found in the gnomAD nor the 1,000 Genomes databases. In silico tools SIFT, PolyPhen‐2, and MutationTaster predicted these variants to be deleterious, probably damaging, and disease causing, respectively (Table S1). Both variants were classified as likely pathogenic according to the ACMG variant classification. The variant c.878A>G occurred in the first position of exon 4 of *HNRNPU*, which is adjacent to the 3′ or splice acceptor site. To determine whether it could affect aberrant splicing, we analyzed the variant c.878A>G in *HNRNPU* using ESE finder 3.0, which rates exonic splicing enhancers using a score.^23^ The score of the *HNRNPU* variant was higher than that of the wild type at the 3′ splice site, meaning that the variant enhances original splicing at that point. However, the variant may create a novel 5′ splice site or splice donor site. A microdeletion on 2q24.3 (Chr2:166740287‐167328969, 588.7‐Kb deletion) in Patient 3 included four genes (*TTC21B*, *SCN1A*, *SCN9A*, and *SCN7A*; Figure 1). Another microdeletion on Xp22.31 (ChrX:6 968 381‐7 268 305, 300‐Kb deletion) in Patient 4 included two genes (*PUDP* and *STS*). Detailed case reports for the patients with identified variants are available in Data S1.

### 3.2 Clinical features

The clinical data from the 29 individuals in this study are summarized in Table 1, Appendix S1, and Table S2. The male‐to‐female ratio was 21:8, and the participants ranged in age from 1 year and 7 months to 28 years old (mean 9 years and 3 months). All patients had MAE, and two had other comorbid disorders. Specifically, Patient 5 had Klinefelter syndrome and Patient 17 had paroxysmal kinesigenic dyskinesia. Patient 26 had juvenile myoclonic epilepsy at the age of 14 years after cessation of AED at 12 years of age. While no patients had a family history of MAE, we found a variety of epilepsies and febrile seizures in three and four families, respectively. Prenatal and perinatal histories were unremarkable, including head circumference at birth. Neurological status and development prior to the onset of seizures were normal except in three patients who exhibited mild developmental delay or autistic behavior and another four patients who experienced febrile seizures between 6 months of age and 3 years and 2 months of age. The first epileptic seizures started between 8 months and 5 years of age. In terms of the first observed seizure type, GTCs were most common, occurring first in 12 patients, and tonic seizures were the second most common, occurring first in 11 patients. At onset, 11 patients had a combination of two or three types of seizure, such as myoclonic seizures and GTCs or myoclonic seizures, GTCs, and atonic seizures.

**TABLE 1:** Clinical characteristics of 29 MAE patients according to seizure prognoses

	Overall (%)	Seizure‐free (%)	Intractable seizures (%)
Total number of patients	29	16	13
Male gender	21 (72)	13 (81)	8 (62)
Neurological signs prior to epileptic seizures	7 (24)	4 (25)	3 (23)
Current age: range, mean ± SD (mo)	19‐336, 111 ± 76	49‐226, 110 ± 61	19‐336, 112 ± 93
Age at seizure onset: range, mean ± SD (mo)	8‐60, 34 ± 13	13‐60, 36 ± 12	8‐60, 32 ± 15
The duration of disease: range, mean ± SD (mo)	7‐312, 77 ± 74	12‐202, 74 ± 61	7‐312, 81 ± 90
Seizure types at onset			
Tonic	11 (38)	5 (31)	6 (46)
Clonic	1 (3)	0 (0)	1 (8)
GTC	12 (41)	7 (44)	5 (38)
Myoclonic	7 (24)	4 (25)	3 (23)
Atonic	3 (10)	3 (19)	0 (0)
Myoclonic‐atonic	4 (14)	3 (19)	1 (8)
Focal	3 (10)	2 (13)	1 (8)
Seizure types during course			
Tonic	1 (3)	0 (0)	1 (8)
Clonic	3 (10)	2 (13)	1 (8)
GTC	2 (7)	0 (0)	2 (15)
Myoclonic	14 (48)	7 (44)	7 (54)
Atonic	7 (24)	3 (19)	4 (31)
Myoclonic‐atonic	3 (10)	1 (6)	2 (15)
Absence (typical or atypical)	13 (45)	6 (38)	7 (54)
NCSE	4 (14)	1 (6)	3 (23)
Focal	1 (3)	0 (0)	1 (8)
EEG findings at onset	28	15	13
Generalized or bisynchronous	17 (61)	11 (73)	6 (46)
Focal or multifocal	5 (18)	2 (13)	3 (23)
No epileptic discharges	6 (21)	2 (13)	4 (17)
EEG findings at the last examination	23	15	8
Generalized or bisynchronous	15 (65)	8 (53)	7 (87)
Focal or multifocal	2 (9)	1 (7)	1 (13)
No epileptic discharges	6 (26)	6 (40)	0 (0)
Development at the last visit			
Severe	3 (10)	0 (0)	3 (23)
Moderate	3 (10)	1 (6)	2 (15)
Mild	7 (24)	4 (25)	3 (23)
Borderline	4 (14)	1 (6)	3 (23)
Normal	12 (41)	10 (63)	2 (15)
Pathogenic variant	4 (14)	2 (13)	2 (15)

Abbreviations: GTC, generalized tonic‐clonic seizure; MAE, myoclonic‐astatic epilepsy; NCSE, nonconvulsive status epilepticus; SD, standard deviation.

Twenty‐seven patients had more than one seizure per day in the period where they experienced the highest number of seizures. Brain MRI findings were unremarkable in all patients except for a mild enlargement of the fourth ventricle in Patient 28. EEG at onset showed that generalized or bisynchronous epileptic discharges were the most common, occurring in 17 patients. All patients exhibited epileptic discharge after onset except for two patients in whom EEG was measured only at 2 years of age. Only six patients, all of whom acquired seizure‐free status, had normalized EEG findings at the last examination. A characteristic rhythmic slow‐wave pattern was observed in 20 patients. The most frequently used AED was VPA, which was the first‐line treatment in 12 patients and the second‐line treatment in four patients, and was efficacious in 23 patients (79%; Table S3). In the study cohort, KD was administered in only two patients and was efficacious in stopping seizures and initiating EEG normalization in one patient. Carbamazepine (CBZ) and ZNS were rarely effective, as reported previously.^3^ Sixteen patients achieved seizure‐free status (seizure‐free group), while the other 13 patients continued to experience seizures (intractable seizure group). Twelve patients had normal development or intelligence, while cognitive dysfunction was borderline in four patients, mild in seven patients, moderate in three patients, and severe in three patients. Motor dysfunction was seen in five patients, but this appeared to be unrelated to MAE in one patient who had exercise‐induced dystonia and one patient who had hemifacial paralysis since birth. Attention‐deficit/hyperactivity disorder (ADHD) was found in 12 patients and autistic spectrum disorder (ASD) in four patients (two patients had both ASD and ADHD).

## 4 DISCUSSION

We collected DNA samples and clinical data including MRI and EEG from 29 patients with MAE. We identified two missense variants in two genes (*SLC6A1* and *HNRNPU*) and two de novo microdeletions in chromosomes 2q24.3 (including *SCN1A*) and Xp22.31 in four patients. *SCN1A* and *SLC6A1* have been reported to be causative for MAE.^9^, ^11^ Patients 1 and 2 had the *SLC6A1* or *HNRPU* variant and showed characteristic EEG findings, such as a theta rhythm during the awake state or a Doose rhythm. While Patient 2, who had the *HNRNPU* variant, showed myoclonic‐atonic seizures, Patient 1, who had the *SLC6A1* variant, had neither myoclonic‐atonic nor myoclonic seizures. *HNRNPU* is causative for intellectual disability with or without dysmorphic features as well as early infantile epileptic encephalopathy‐54 (EIEE54: OMIM #617391),^24^, ^25^ although no patients with MAE have been reported to have any variant in *HNRNPU* to date. *HNRNPU*, heterogeneous nuclear ribonucleoprotein U, is located on human chromosome 1q44 and encodes an RNA binding protein involved in pre‐mRNA processing.^26^ A microdeletion at 1q44 produces phenotypes similar to those associated with an intragenic variant of *HNRNPU*. Haploinsufficiency is suspected to be the major pathogenic mechanism for *HNRNPU* variants. Patients with *HNRNPU* variants show moderate‐to‐severe intellectual disability, as well as epileptic seizures that are mainly generalized such as tonic, atonic, and absence seizures. These usually start before 5 years of age, for instance, around 12 months of age in 88% of patients, and 62% of patients experience febrile seizures.^24^, ^25^ Patient 2 in this study, who had an *HNRNPU* variant, had febrile seizures at 15 months prior to generalized epileptic seizures at 5 years of age. However, she had neither intellectual disabilities nor dysmorphic features. Further investigations are required to confirm the causal role of *HNRNPU* in MAE.

Patient 4 showed a microdeletion at Xp22.31 that included two genes, *PUDP* and *STS. PUDP* itself has not been linked to disorders in humans, and there are no obvious clinical differences between STS‐deficient patients with and without PUDP deletion.^27^ Thus, it is unlikely to be harmful. *STS*‐encoding steroid sulfatase is a causative gene for X‐linked ichthyosis, which is characterized by ichthyosis from birth and cognitive behavioral features such as intellectual disability, ADHD, and ASD.^28^ Patient 4 showed no signs of typical ichthyosis, but he did have dry skin, which suggested mild skin lesions. Interestingly, four of the 30 patients (13%) with X‐linked ichthyosis had an epilepsy onset between 10 months and 12 years of age.^28^ Thus, *STS* may be associated with molecular pathogenesis in MAE, albeit further investigations are required.

Fourteen percent of the participants in this study had gene variants (4/29). While this ratio varies from 3% to 41% in similar studies for MAE (in which the original definition by Doose was applied),^10^, ^29^ the frequency of gene variants identified in the present study is comparable to that in a recent study of 77 patients with MAE (in which the recent ILAE definition was applied), in which a molecular diagnosis was determined for six of 59 patients (10%).^30^ Variants of *SLC6A1, SLC2A1,* and *SCN1A* are relatively common in patients with MAE, with reported variant rates of 4% (6/160),^11^ 4.8% (4/84),^9^ and 5% (1/20),^31^ respectively. Although the variant rate is inconstant, the underlying causes of MAE show genetic heterogeneity. Whole‐exome sequencing or target capture sequencing is recommended to uncover causative genes in patients with MAE.

The ratio of patients who achieved seizure‐free status in previous studies has varied, with rates such as 32%, 58%, 67%, and 68%, in studies that used the original definition by Doose,^1^, ^6^, ^32^, ^33^ and 38% in studies in which the recent ILAE definition was applied.^18^ Because we recruited patients with the aim of conducting gene analysis, it is possible that our study cohort included a higher number of patients with more severe or intractable conditions. However, 55% of the patients in this study were seizure‐free, so the seizure prognoses in this study were comparable to those in previous studies. The pathogenic variant‐detection ratio was similar between the intractable (15%) and seizure‐free groups (13%). Although patients with epileptic encephalopathies have a higher rate of pathogenic variants compared with other epilepsy patients,^34^ analyses of causative genes might be valuable even in patients for which medication is able to effectively relieve seizures.

Interestingly, the frequency of febrile seizures prior to epileptic seizures (4/29, 14%) was higher in our study group compared with that in the general Japanese population (7%–11%).^35^ Febrile seizures in patients with MAE (eg, 8/72, 11%; 5/57, 14%, according to the recent ILAE definition and the original definition by Doose, respectively) are associated with a higher morbidity rate than that in the general population.^18^, ^36^ Two out of four patients with febrile seizures in our cohort had a gene variant, such as *HNRNPU*, or a microdeletion at Xp22.31. Unknown causative genes for febrile seizures might modulate the incidence of MAE.

The semiology of seizures in our group of MAE patients had a pattern that was similar to that previously reported, that is, myoclonic, atonic, myoclonic‐atonic, tonic, GTC, and atypical absence seizures.^5^ Myoclonic seizures and absence (including typical and atypical) seizures were more frequently seen in the patients in the intractable group compared with those in the seizure‐free group (85% vs 63%, 54% vs 31%, respectively), which suggests that myoclonic seizures and absence seizures may be intractable factors. The frequency of myoclonic‐atonic seizures, which is a requirement for the recent ILAE definition of MAE, at onset seems to be higher in patients with gene variants compared with patients with no variant (50% vs 8%). Thus, this frequency might be a clue regarding the presence of gene variants in patients with MAE.

A recent study reinforced the difficulty in distinguishing between MAE and Lennox‐Gastaut syndrome, which is characterized by classical traits of tonic seizures, paroxysmal fast activity, and slow spike‐and‐wave activity.^18^ In the present study, while 12 of the 29 (41%) patients showed tonic seizures, none exhibited slow spike‐and‐wave EEG activity because we excluded the patients with Lennox‐Gastaut syndrome based on EEG findings.

Of the 29 patients, 12 (41%) had normal development or normal IQ. This is similar to that previously reported for cohorts of MAE patients (IQ > 75, 6%–59%).^5^ As expected, the seizure‐free group showed better development and/or IQ than the intractable seizure group (normal development or IQ, 56% vs 23%), and motor dysfunction was rarely seen in either group.

There are several limitations to the present study. First, because MAE is a rare form of epilepsy, we could not examine a large number of cases. This affected our ability to test the statistical significance of our results. Second, the pathogenicity of each variant in this study was not confirmed by functional studies using cell lines or animal models. Third, the ratio of pathogenic variant‐positive cases (14%) was not sufficient to determine the genotype‐phenotype relationship in this study. Previous studies reported a pathogenic variant ratio of 3%–41% in a MAE cohort.^10^, ^29^ Despite these limitations, this study adds to the existing literature as a comprehensive evaluation of the clinical and genetic features of a cohort of MAE patients.

The MAE patients in the present study showed genetic heterogeneity. We identified *HNRNPU* and *STS* as possible novel candidates for causative genes for MAE. To further examine genetic predisposition for MAE, mutation screening with whole‐exome sequencing or target capture sequencing is highly recommended.

## DISCLOSURE OF CONFLICT OF INTERESTS

None of the authors has any conflict of interest to disclose. We confirm that we have read the Journal's position on issues involved in ethical publication and affirm that this report is consistent with those guidelines.

## Supporting information

Supplementary Material

## Data Availability

The data sets generated during and/or analyzed during the current study are available from the corresponding author upon reasonable request.
