# Supplementary material for: Clinical and genetic characteristics of patients with Doose syndrome
Source: Epilepsia Open. 2020 Jul 23;5(3):442–50. doi: 10.1002/epi4.12417 (PMC7469791; doi:10.1002/epi4.12417)
Supplement: Supplementary file 1 — Supplementary Material [file EPI4-5-442-s001.zip › epi412417-sup-0001-AppendixS1.docx]

Appendix S1. Case reports describing patients with MAE in whom mutations were identified.

Patient 1 (*SLC6A1* c.739C>G, p.(Pro247Ala))

Patient 1 was born spontaneously with no asphyxia as the second child of unrelated healthy parents. Her parents noticed recurrent falling-down behavior at the age of 3 years. She showed frequent falling-down with eye-closure or sudden astatic episodes leading to a diagnosis of atonic seizures at 4 years of age. EEG showed continuous bilateral occipital spike-and-slow waves and diffuse spike-and-slow wave bursts twice per minute. Although her seizures were refractory to several anti-epileptic drugs, nitrazepam finally stopped her seizures at 7 years of age. She showed head control at 3 months, sitting alone at 10 months, walking alone at 16 months, and two-phrase speech at 24 months. She showed evidence of a mild intellectual disability. At the age of 16 years, a central-dominant 6–7 Hz theta rhythm appeared during the awake state with sudden diffuse slow-wave bursts.

Patient 2 (*HNRNPU* c.878A>G, p.(Tyr293Cys))

Patient 2 was born spontaneously after an uneventful pregnancy with no asphyxia as the child of unrelated healthy parents. Simple febrile convulsions occurred at 1 year and 3 months old. She experienced myoclonic seizures at 5 years of age followed by afebrile generalized clonic convulsions at 5 years and 6 months. She was treated with multiple AEDs, such as VPA, CZP, and levetiracetam (LEV), but these did not reduce the frequency of atypical absence seizures, which occurred three times a day. She showed head control at 3 months, sitting alone at 9 months, walking alone at 13 months, meaningful words at 18 months, and two-phrase speech at 24 months. Her intelligence quotient was 93 at 5 years and 9 months of age, and intellectual regression was suspected because of a decrease in memory retention. EEG recordings showed frontocentral-dominant diffuse slow-wave bursts or frequent spike-and-slow waves at 5 years and 6 months followed by bilateral frontoparietal-predominant theta rhythms during the awake state with frequent diffuse (poly) spike-and-slow waves during both the awake and asleep states at 6 years and 2 months. At 6 years and 10 months, high doses of ethosuximide led to the disappearance of her seizures and epileptic discharges with the exception of frontal slow wave activities during the awake state, as revealed by EEG.

Patient 3 (2q24.3, 588.7-Kb deletion including *SCN1A*)

Patient 3 was born by cesarean section at full term with no asphyxia as the second child of unrelated healthy parents. Her maternal uncle had a history of febrile seizure. She had generalized tonic–clonic convulsions (GTCs) and myoclonic-atonic seizures during the awake state at 8 months of age. While CBZ exacerbated GTCs, VPA and CBZ reduced the frequency of GTCs. Myoclonic–atonic seizures disappeared with LEV. She continued to have hourly focal impaired awareness seizures and daily generalized tonic–clonic seizures. She engaged in social smiles at 3 months, head control at 3 to 4 months, and walking alone and meaningful words at 15 months. Her body height was 78 cm (-1.43 SD) and weight was 11.3 kg (+0.9 SD) at 1 year and 9 months of age. Her development was borderline.

Patient 4 (300-Kb deletion at Xp22.31)

Patient 4 was born spontaneously at full term with no asphyxia as the child of unrelated healthy parents. At the age of 2 years, he experienced a 25-minute episode of hemiclonic convulsions induced by a high fever. EEG showed epileptic discharges at the temporal and occipital regions. Four months later, he experienced afebrile hemiclonic convulsions, leading to the decision to commence VPA administration. At the age of 5 years, atonic seizures followed by tonic seizure for 1 minute mainly occurred during the sleep state in the early morning. He had a microdeletion at chromosome Xp22.31, which contains the STS (steroid sulfatase) gene (Figure 1) for which variants are causative for X-linked ichthyosis. While he had no typical dermatological signs suggesting ichthyosis, he had dry skin, particularly on the abdomen.
