# Supplementary material for: Clinical and genetic characteristics of patients with Doose syndrome
Source: Epilepsia Open. 2020 Jul 23;5(3):442–50. doi: 10.1002/epi4.12417 (PMC7469791; doi:10.1002/epi4.12417)
Supplement: Supplementary file 1 — Supplementary Material [file EPI4-5-442-s001.zip › epi412417-sup-0003-TableS1.docx]

**Supplemental Table 1. Prediction of variant pathogenicity**

| Subject | Gene | Variant | Origin | dbSNP | HGMD | gnomAD | PolyPhen-2 | SIFT | CADD | PROVEAN | PANTHER | ACMG variant classification |
| --- | --- | --- | --- | --- | --- | --- | --- | --- | --- | --- | --- | --- |
| Patient 1 | *SLC6A1* | NM_003042.3:c.739C>G, p.(Pro247Ala) | de novo | - | - | - | 1 (probably damaging) | 0 (deleterious) | 25.2 | -7.454 (deleterious) | -8.42951 (deleterious) | Likely pathogenic (PS2, PM2, PP3) |
| Patient 2 | *HNRNPU* | NM_031844.2:c.878A>G, p.(Tyr293Cys) (*3) | de novo | - | - | - | 0.999 (probably damaging) | 0 (deleterious) | 34 | -7.519 (deleterious) | -4.0999 (deleterious) | Likely pathogenic (PS2, PM2, PP3) |
