# Supplementary material for: Clinical and genetic characteristics of patients with Doose syndrome
Source: Epilepsia Open. 2020 Jul 23;5(3):442–50. doi: 10.1002/epi4.12417 (PMC7469791; doi:10.1002/epi4.12417)
Supplement: Supplementary file 1 — Supplementary Material [file EPI4-5-442-s001.zip › epi412417-sup-0004-TableS2.docx]

**Supplemental Table 2. Summary of clinical features in MAE patients for whom we identified pathogenic variants.**

| **Patient** | **Variant** | **Age, Sex** | **Familial seizures** | **Neurological signs prior to epileptic seizures** | **Onset and types of the first seizure** | **Onset and types of other seizures** | **Peak frequency of seizures** | **EEG findings** | **Effective AEDs** | **Ineffective AEDs** | **Seizure prognosis** | **Development or intelligence** | **Other comorbidities** |
| --- | --- | --- | --- | --- | --- | --- | --- | --- | --- | --- | --- | --- | --- |
| **1** | *SLC6A1*c.739C>G, p.(Pro247Ala) | 16y, F | No | Speech delay at 2y and liable to fall down at 3y | 4y0m, atonic, brief impaired aware­ness | No | 2-4/w | Bisynchronous occipital SpW and generalized SpW burst during seizures at 4y, then 6Hz theta waves during awake state and SpW burst during seizures at 16y | VPA, CZP, NZP, LTG | ESM, ZNS | Seizure-free | Mild ID, IQ 61 | No |
| **2** | *HNRNPU*c.878A>G, p.(Tyr293Cys) or aberrant splicing | 6y, F | Paternal cousin: febrile seizures | Febrile seizure at 1y3m | 5y, myo­clonic, myoclonic-atonic | 5y6m, clonic, atypical absence | 8/day | Frontocentral-dominant diffuse slow-wave bursts with frequent SpW at 5y6m, frontoparietal theta rhythm during awake state and frequent generalized SpW and poly SpW at 6y2m, frontal dominant diffuse slow wave during awake state at 6y10m | ESM | VPA, CZP, LEV | Seizure-free | Normal, DQ 93 | No |
| **3** | Deletion at 2q24.3 including *SCN1A* | 1y9m, F | Maternal uncle: febrile seizures | No | 8m, GTC, myclonic-atonic | 1y, ab­sence | 10/day | Normal at the onset, central and parietal 4-5Hz diffuse theta waves at 1y7m, right frontal fast waves evolving to generalized 2.5-3 Hz SpW burst during seizures | VPA, PB, LEV | ACTH, CLB, ESM, CBZ, PHT | Intractable | Borderline | No |
| **4** | Deletion at Xp22.31 including *STS* | 6y9m, M | No | Febrile seizures at 2y0m | 2y4m, hemiclonic | 5y, tonic, atonic | 1/day | Right focal spikes at 2y | VPA | CZP | Intractable | Borderline | Left-side clumsi­ness |
| 5 | No | 1y7m, M | Paternal grandmother: epilepsy | Delayed head control at 6 m | 0y8m, tonic | 1y4m, atypical absence, myoclonic | 45/day | Generalized polySpW during sleep state at 1y | VPA | CLB, LTG | Intractable | Borderline | ADHD |
| 6 | No | 2y9m, M | No | No | 2y, tonic | 2y, clonic | 20-30/day | Diffuse 3-4Hz slow waves with generalized spikes during myoclonic seizure at 2y8m | VPA,CZP,LTG |  | Intractable | Normal | ADHD |
| 7 | No | 5y0m, M | No | No | 2y10m, myoclonic, GTC | No | 2-10/day | Normal at 2y10m | VPA, PB, CLB |  | Seizure-free | Normal | No |
| 8 | No | 3y11m, M | No | No | 2y9m, myoclonic, GTC | No | 5-6/day | Normal at 2y9m | VPA | CZP, LTG | Intractable | Mild delay, DQ 63 | No |
| 9 | No | 5y3m, M | No | No | 2y8m, myoclonic, GTC, atonic | No | 2-4/day | Parietal 5Hz theta rhythm during awake state with 2-2.5Hz SpW during sleep state and generalized SpW during myoclonic seizures at 2y10m, then normalized at 4y8m | VPA, CZP |  | Seizure-free | Normal | No |
| 10 | No | 3y8m, M | No | No | 3y1m, tonic | 3y2m, myoclonic, myoclonic-atonic | 2-5/day | Generalized SpW with diffuse 4-6Hz theta wave at 3y | KD, VPA, CLB | LTG, LEV | Intractable | Mild ID, IQ 65 | Mild tremor, ADHD, aggressive |
| 11 | No | 4y1m, M | Mother's two cousins: juvenile-onset epilepsy | No | 3y1m, tonic | 3y4m, atonic | 10-15/day | Generalized SpW at 3y, diffuse theta burst with bisynchronous frontopolar spikes at 3y6m | VPA |  | Seizure-free | Normal | No |
| 12 | No | 4y3m, M | No | 2y- ASD | 3y, myoclonic-atonic | No | 1-17/day | Diffuse high amplitude slow wave bursts and frontal-dominant bisynchronous SpW at 4y4m | VPA |  | Seizure-free | Normal, DQ 89 | ASD |
| 13 | No | 4y5m, M | No | No | 2y10m, myoclonic | 3y6m, atonic | 3-10/day | Generalized (poly) SpW at 2y10m, then normalized at 4y5m | LTG | ZNS, VPA, LEV, CLB | Seizure-free | Normal, IQ 90 | No |
| 14 | No | 4y7m, M | No | No | 2y10m, GTC | 3y0m, myoclonic, NCSE, atypical absence | 20-30/day | Diffuse high amplitude theta waves with bisynchronous frontal-dominant SpW frequently at 3y and 3y4m | PHT, LEV, K/NaBr, PMP, MDL | VPA, CLB, CZP, PB, ESM, TPM, LTG, LCM | Seizure-free | Mild delay | No |
| 15 | No | 4y11m, F | No | No | 3y1m, GTC | 3y2m, GTC, myoclonic | 10/day | Normal at 3y2m, diffuse 5-6Hz theta bursts during awake state and bisynchronous frontal 1-3Hz poly SpW during sleep state at 4y11m | LEV | VPA, CZP, CLB | Intractable | Normal | No |
| 16 | No | 15y1m, M | Father and elder sister: febrile seizures | No | 3y1m, GTC, tonic | 3y4m, myoclonic, atypical absence | several times/day | Generalized SpW at 3y1m, normal at 8 y | ZNS, VPA, CZP, ACTH, ESM, LTG, LEV | BZPs, CBZ, KD | Seizure-free | Moderate delay, IQ 49 | Mild ataxia, ADHD |
| 17 | No | 5y6m, M | No | No | 1y1m, impaired awareness | 2y3m, clonic, myoclonic | several times/day | Continuous rhythmic theta activity at right hemisphere at 2y3m. Frontal spikes and diffuse 4-5Hz theta rhythm during interictal awake state and generalized high amplitude SpW during myoclonic seizures at 4y | VPA, CLB | CBZ, CZP, LEV | Seizure-free | Mild delay, DQ 58 | Exercise-induced dystonia |
| 18 | No | 16y3m, F | Paternal grandmother: afebrile convulsion in childhood | Febrile seizures at 7 m | 2y11m, tonic | 3y, myoclonic, atonic, atypical absence, NCSE | 1-2/day | Generalized (poly) SpW during sleep state with parieto-occipital theta waves during awake state at 6y, 3-4Hz generalized SpW and bisynchronous frontoparietal SpW at 16y | VPA, CZP,ESM, LTG | BZPs, TPM | Intractable | Moderate delay, IQ 40 | ADHD |
| 19 | No | 10y8m, M | No | No | 1y11m, tonic | 2y2m, atonic, myoclonic, atypical absence, NCSE, FIAS | 9/day | Central theta rhythm at 2y2m, diffuse 2-3Hz slow waves with bisynchronous frontal spikes at 2y3m, generalized irregular poly SpW at 10y | CZP, ESM, LEV | ZNS, VPA, CLB, LTG, PSL, TPM, RFN | Intractable | Severe delay, DQ 34 | ADHD, ASD |
| 20 | No | 8y2m, M | No | No | 2y0m, GTC | 2y1m, myoclonic | frequent/day | Normal at 2y followed by generalized epileptic discharges until 7y11m | VPA, LTG | CBZ, worsened | Seizure-free | Normal, IQ 98 | ADHD |
| 21 | No | 11y8m, M | No | Febrile seizures at 3y2m | 3y2m, tonic, GTC | 3y3m, myoclonic, atypical absence, GTC, tonic | 10/day | Symmetrical 6-8Hz 50-100 Hz rhythm with bisynchronous frontal 1.5Hz SpW at 3y3m, then normalized at 10y5m | VPA, CLB, ESM, LEV | ZNS | Seizure-free | Normal, DQ 88 | ADHD |
| 22 | No | 8y10m, M | No | No | 2y9m, GTC | 4y5m, myoclonic-atonic | 50/day | Left-dominant multifocal sharp-and-slow waves at 3y, left temporal sharp wave at 8y | Vitamin B6, VPA, LTG | CBZ, CLB | Seizure-free | Normal | ADHD |
| 23 | No | 10y, F | No | No | 5y, myoclonic, GTC | 9y, myoclonic-atonic | >1/day | Generalized SpW with parietooccipital 150-200μV theta rhythm at 5y2m, frontal-dominant high amplitude theta rhythm and generalized high-amplitude polySpW at 10y | VPA, TPM | CZP, CLB, LTG, LEV, steroid | Intractable | Mild ID, IQ 54 | No |
| 24 | No | 15y4m, M | No | No | 2y11m, tonic, atonic | 3y3m, myoclonic, atypical absence | 4-5/day | Bisynchronous parietal 6-7Hz theta wave with no epileptic discharges during awake state at 2y11m, then normalized at 10y | VPA, CZP, ESM |  | Seizure-free | Mild ID, IQ 66 | Left facial paralysis since birth, ADHD |
| 25 | No | 15y4m, M | Father and brother: febrile seizure | No | 3y3m, clonic, GTC | 3y4m, myoclonic, atypical absence, atonic | 7/day | Left or right frontocentral SpW at 3y4m, then bisynchronous parietal 3-5Hz slow wave bursts and bisynchronous frontocentral 5-7Hz theta wave during awake state with frequent bisynchronous frontopolar SpW and propagated to bisynchronous parietal area at 12y4m | VPA, PB, ESM | ZNS, CBZ, CLB, CZP, LEV, LTG, PER | Intractable | Severe ID, IQ 27 | Dysphagia, ADHD, ASD |
| 26 | No | 18y10m, M | No | No | 2y0m, tonic | 2y0m, atypical absence, myoclonic, atonic | >100/day | Generalized poly SpW at 2 y, normalized at 13 y, frontal-dominant generalized SpW at 14y8m | VPA, CZP | PB | Seizure-free at 3 years of age and JME at 14 years | Normal |  |
| 27 | No | 14y, F | No | No | 5y0m, myoclonic-atonic | No | several times/day | 3-4 Hz diffuse delta-theta burst and generalized SpW at 5y, then normalized at 11y | ESM, NZP | VPA, CBZ, CZP | Seizure-free | Borderline | No |
| 28 | No | 16y, M | No | No | 4y6m, tonic | 4y6m, atypical absence, myoclonic, NCSE | >20/day | Diffuse theta wave bursts during awake state with no epileptic discharges at 4y6m, continuous theta wave during light sleep and generalized spikes, SpW and sharp-and-slow waves at 13y | VPA, CZP, CLB, RFN, AZA, PIR | NZP, PB, TPM, LTG, KBr/NaBr, PRM | Intractable | Moderate ID | ADHD |
| 29 | No | 28y, M | No | No | 2y, myoclonic | 2y9m, atypical absence, tonic-clonic | >1/day | Right central focal spikes with 3-4 Hz slow wave dysrhythmia at 2y, right-predominant frontal spikes during sleep state at 20y | VPA, CZP, PB, PHT, LTG, LEV | NZP, ESM, ZNS, TPM | Intractable | Severe delay, DQ 34 | ASD |
| Abbreviations: ACTH, adrenocorticotropic hormone; ADHD, attention deficit hyperactivity disorder; AEDs, anti-epileptic drugs; ASD, autism spectrum disorder; AZA, acetazolamide; BZPs, benzodiazepines; BW, body weight; CBZ, carbamazepine; CLB, clobazam; CZP, clonazepam; DQ, developmental quotient; EEG, electroencephalogram; ESM, ethosuximide; F, female; FIAS, focal impaired awareness seizures; GBP, gabapentin; GTC, generalized tonic-clonic; ID, intellectual disability; IGE, idiopathic generalized epilepsy; IQ, intelligence quotient; JME, juvenile myoclonic epilepsy; K/NaBr, potassium/sodium bromide; KD, ketogenic diet; LCM, lacosamide; LEV, levetiracetam; LTG, lamotrigine; m, month(s); M, male; MAE, myoclonic astatic epilepsy; NA, not available; NCSE, non-convulsive status epilepticus; NZP, nitrazepam; PB, phenobarbital; PER, perampanel; PHT, phenytoin; PIR, piracetam; PRM, primidone; PSL, prednisolone; RFN, rufinamide; SB, suppression burst; SpW, spike-and-wave complex; TPM, topiramate; VPA, valproic acid; w, week(s); y, year(s); ZNS, zonisamide | | | | | | | | | | | | | |
