# Supplementary material for: Clinical and genetic characteristics of patients with Doose syndrome
Source: Epilepsia Open. 2020 Jul 23;5(3):442–50. doi: 10.1002/epi4.12417 (PMC7469791; doi:10.1002/epi4.12417)
Supplement: Supplementary file 1 — Supplementary Material [file EPI4-5-442-s001.zip › epi412417-sup-0005-TableS3.docx]

**Supplemental Table 3. Efficacy of anti-epileptic drugs in patients with MAE.**

| Antiepileptic drug | Effective  (person) | Non-effective  (person) | Total  (person) | Ratio of effective patients in total number of patients |
| --- | --- | --- | --- | --- |
| VPA | 23 | 6 | 29 | 0.79 |
| CZP | 10 | 8 | 18 | 0.56 |
| LTG | 8 | 6 | 14 | 0.57 |
| CLB | 5 | 8 | 13 | 0.38 |
| LEV | 7 | 6 | 13 | 0.54 |
| ESM | 8 | 4 | 12 | 0.67 |
| PB | 4 | 3 | 7 | 0.57 |
| ZNS | 1 | 7 | 8 | 0.13 |
| CBZ | 0 | 7 | 7 | 0.00 |
| TPM | 1 | 5 | 6 | 0.17 |
| PHT | 2 | 1 | 3 | 0.67 |
| NZP | 2 | 2 | 4 | 0.50 |
| KD | 1 | 1 | 2 | 0.50 |
| KBr or NaBr | 1 | 1 | 2 | 0.50 |
| ACTH | 1 | 1 | 2 | 0.50 |
| Rufinamide | 1 | 1 | 2 | 0.50 |
| BZPs | 0 | 2 | 2 | 0.00 |
| PSL | 0 | 2 | 2 | 0.00 |
| Piracetam | 1 | 0 | 1 | 1.00 |
| Vit.B6 | 1 | 0 | 1 | 1.00 |
| PMP | 1 | 0 | 1 | 1.00 |
| MDZ div | 1 | 0 | 1 | 1.00 |
| PER | 0 | 1 | 1 | 0.00 |
| Primidone | 0 | 1 | 1 | 0.00 |
| LCM | 0 | 1 | 1 | 0.00 |

Abbreviations: ACTH, adrenocorticotropic hormone; AZA, acetazolamide; BZPs, benzodiazepines; CBZ, carbamazepine; CLB, clobazam; CZP, clonazepam; ESM, ethosuximide; GBP, gabapentin; KBr, potassium bromide; KD, ketogenic diet; LCM, lacosamide; LEV, levetiracetam; LTG, lamotrigine; m, month(s); MAE, myoclonic astatic epilepsy; MDZ, midazolam; NaBr, sodium bromide; NZP, nitrazepam; PB, phenobarbital; PER, perampanel; PHT, phenytoin; PIR, piracetam; PSL, prednisolone; TPM, topiramate; VPA, valproic acid; ZNS, zonisamide
