# Supplementary figures and images for: Clinical and genetic characteristics of patients with Doose syndrome
Source: Epilepsia Open. 2020 Jul 23;5(3):442–50. doi: 10.1002/epi4.12417 (PMC7469791; doi:10.1002/epi4.12417)

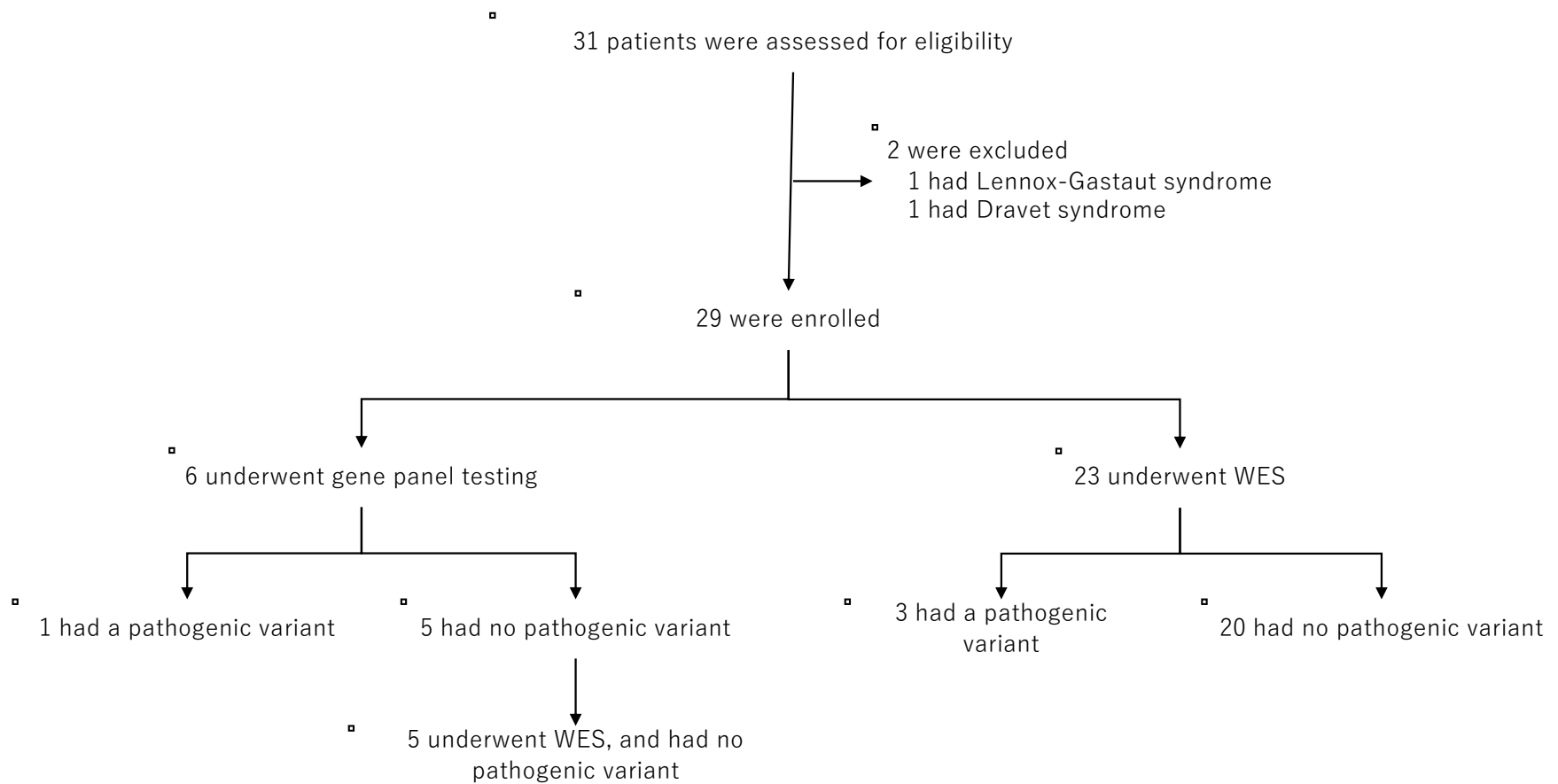

Figure S1. Flowchart showing the recruitment of MAE patients who participated in this study

Supplement: Supplementary file 1 — Supplementary Material [file EPI4-5-442-s001.zip › epi412417-sup-0002-FigS1.pdf]
